# Atypical Amniotic Fluid Embolism Managed with a Novel Therapeutic Regimen

**DOI:** 10.1155/2017/8458375

**Published:** 2017-12-21

**Authors:** Shadi Rezai, Alexander C. Hughes, Tracy B. Larsen, Paul N. Fuller, Cassandra E. Henderson

**Affiliations:** ^1^Department of Obstetrics and Gynecology, Kaiser Permanente Southern California, 1200 Discovery Drive, Bakersfield, Kern County, CA 93309, USA; ^2^School of Medicine, St. George's University, St. George's, Grenada; ^3^Department of Anesthesiology, Adventist Health Bakersfield, 2615 Chester Avenue, Bakersfield, CA 93301, USA; ^4^Department of Obstetrics and Gynecology, Lincoln Medical and Mental Health Center, 234 East 149th Street, Bronx, NY 10451, USA

## Abstract

Amniotic fluid embolism (AFE) is the second leading cause of maternal mortality in the USA with an incidence of 1 : 15,200 births. The case fatality rate and perinatal mortality associated with AFE are 13–30% and 9–44%, respectively. This rare but devastating complication can be difficult to diagnose as many of the early signs and symptoms are nonspecific. Compounding this diagnostic challenge is a lack of effective treatment regimens which to date are mostly supportive. We present the case of a 26-year-old woman who suffered from suspected AFE and was successfully treated with the novel regimen of Atropine, Ondansetron, and Ketorolac (A-OK). The authors acknowledge that this case does not meet the new criteria proposed, by Clark in 2016, but feel that it is important to share this case report, due to dramatic patient response to the provided supportive therapy presented in this case report. We hope this case report will prompt further research into this novel approach to treating AFE with Atropine, Ondansetron, and Ketorolac.

## 1. Introduction

Amniotic fluid embolism (AFE) is a rare, unpredictable, and potentially devastating complication of childbirth, in which amniotic fluid, fetal cells, hair, or other types of debris enter into the maternal pulmonary circulation, causing cardiovascular collapse [1, 2]. The incidence of AFE ranges from 1 : 15,200 to 1 : 53,800 [3–5]. AFE is the second leading direct cause of maternal death in the USA and Europe [3–6]. Conde-Agudelo and Romero found the percent total maternal deaths due to AFE to be 13.7%, slightly higher than the previous widely held 10% [5].

Early recognition and initiation of treatment of AFE are essential to increase the likelihood of patient survival [1, 7]. This can be a challenge, as AFE is a diagnosis of exclusion with no universal pathological or serological markers [1, 8, 9]. Transesophageal echocardiography (TEE) can be used to determine cardiac dysfunction due to pulmonary hypertension but may not be widely available on obstetric units [7, 10]. AFE is traditionally diagnosed clinically, in a woman early during labor with ruptured membranes, by a trio of symptoms: acute respiratory distress, cardiovascular collapse, and coagulopathy [3–5, 8, 9, 11]. Other symptoms include hypotension, frothing from the mouth, fetal heart rate abnormalities, loss of consciousness, bleeding, uterine atony, and seizure like activity [8, 12]. However, as a diagnosis of exclusion, the AFE triad is neither sensitive nor specific and should be considered once other diagnoses have been ruled out. Clark has recently proposed diagnostic criteria for AFE case report in order to prevent over reporting, but the Society for Maternal Fetal Medicine (SMFM) continues to support the current clinical diagnosis [11, 13].

With a greater understanding of the pathophysiology of AFE, new therapies have shown potential [4, 5]. Copper et al. have reported “that antiserotonin, antithromboxane, and vagolytic therapy” were the mechanisms for the restoration of a patients' circulation and led to successful resuscitation [14]. We present a similar case of a 26-year-old woman with suspected AFE who was successfully managed with traditional therapy and a novel regimen of Atropine, Ondansetron, and Ketorolac (A-OK) [4, 14].

## 2. Case History

A 26-year-old Hispanic female, G2P1001, at 38  1/7 weeks of gestation complicated by obesity (BMI of 41) and gestational diabetes (GDM2) presented to the emergency room complaining of shortness of breath for approximately 8 hours. On exam she was noted to have a fever (102.2 degrees Fahrenheit or 39.0 degrees Celsius), blood pressure of 119/73 mm Hg, maternal tachycardia (144 beats per minute (BPM)), tachypnea (24 breaths/minute), oxygen saturation of 97%, and fetal tachycardia (211 BPM). The cervical exam was 1 cm cervical dilatation, zero percent effacement, long and posterior position fetus in vertex presentation, and intact amniotic fluid membrane (i.e., 1 cm/long/posterior, vertex, and intact). Urine toxicology screen was negative. Intravenous hydration was initiated and the patient was started on broad spectrum antibiotics for the empirical treatment of sepsis (Piperacillin/Tazobactam, Vancomycin). The patient underwent stat primary low transverse cesarean delivery due to nonreactive tracing and fetal tachycardia with minimal variability (category 2 tracing) under general endotracheal intubation anesthesia with rapid sequence intubation. A total of 200 mg of Propofol with 100 mg of succinylcholine were rapidly infused, and blood pressure at that time was 80/40 mg Hg. A size 7.0 endotracheal tube (ETT) was placed under direct laryngoscopy with clear view of tube passing the vocal cords. Intubation was atraumatic with a grade 1 view. Endotracheal tube was taped and secured at 21 cm. Bilateral breath sounds were obtained by auscultation and positive CO_2_ per anesthesia monitor were used to confirm placement.

Patient delivered an infant with Apgar of 9 and 9 in one and five minutes, respectively, with meconium amniotic fluid. Immediately after delivery of the infant and before the extraction of placenta, the patient's heart rate remained in 140 beats per minute (BPM), but the patient's oxygen saturation decreased to 72%, blood pressure lowered to 72/48 mm Hg, and end-tidal CO2 (ETCO2) as per the anesthesia monitor fell from 32 to 0 mm Hg. Normal reference range for ETCO2 is between 35 and 45 mm Hg. The anesthesia equipment was rapidly checked to confirm that there was no equipment leak or disconnection as the phenylephrine IVP was administered with an initial dose of 200 mcg and repeated several times for a total of 1800 mcg (see Table 1 for summary). The patient was evaluated by the obstetrics team for hysterotomy extensions, lacerations, or uncontrolled bleeding (suggestive of DIC) which was found to be negative.

The anesthesia team initiated A-OK AFE protocol within one minute of onset of the listed symptoms. A-OK consisting of 0.2 mg Atropine, 8 mg Ondansetron, and 15 mg Ketorolac were all given as intravenous push. Within 2-3 minutes the patient's oxygen saturation recovered to 97% and blood pressure increased to 138/68 mm Hg, CO2 returned per monitor to 32 mm Hg, but tachycardia remained with a heart rate of approximately 140 BPM (see Figure 2 and Table 2). Patient's oxygen saturation and blood pressure responded to medical management by the anesthesia team with intravenous fluids, Atropine, Ondansetron, and Ketorolac. As the patient responded to medical management, her blood pressures and oxygen saturation quickly improved. To treat uterine atony and intraoperative hemorrhage, the patient received 50 units of Oxytocin and 2 doses of Carboprost, 3 units of packed red blood cells (PRBCs), 1 unit of fresh frozen plasma (FFP), and 3,500 ml intravenous fluid. She had 2,000 ml estimated blood loss (EBL).

Once stabilized, she remained intubated and was transported to the intensive care unit (ICU) for further monitoring. Postoperative chest computed tomography (CT) scan with and without contrast did not show any evidence of pulmonary embolism (PE) but showed bibasilar atelectasis with no evidence of definite consolidation and/or pneumonia. Lower extremity Doppler ultrasound showed negative results for deep venous thrombosis (DVT). Intraoperative and postoperative laboratory blood works were also negative for DIC with PT, PTT, and INR within normal limits. Blood culture, urine culture, and sputum cultures were taken and later found to be negative. Chest X-ray was done that showed no acute pathology. Placental pathology was negative for chorioamnionitis, placental abruption, and retroplacental hematoma.

The patient was extubated on postoperative day one. Antibiotics were switched to Cefazolin. The patient remained afebrile and asymptomatic with stable vital signs. The patient and newborn had an uneventful postoperative recovery course and were both discharged on postoperative day 3 with a follow-up appointment at our clinic.

## 3. Discussion

The case described is a woman, with no known risk factors for AFE (summarized in Table 3), presenting to the emergency room in distress with subsequent rapid decomposition after delivery [17, 18]. The clinical diagnosis of AFE was made and differential diagnoses were ruled out. The patient was managed with traditional cardiovascular support and administration of PRBCs and FFP while the anesthesia team initiated the A-OK therapy. Shortly after A-OK therapy and phenylephrine, the patient experienced a rapid reversal of symptoms and stabilization [7, 19].

AFE has traditionally been a diagnosis of exclusion made in an emergency situation [11]. Recently, Clark et al. have proposed diagnostic criteria for scientific research but this has not yet been widely adopted [13, 20]. The Society for Maternal Fetal Medicine (SFMFM) which continues to endorse AFE is a clinical diagnosis with an emphasis on maintaining cardiovascular function and hemodynamic stability [11]. In this case the criteria outlined in Clark et al. 2016 (see Table 4) were not met. The main exclusion criteria, from Clark et al.'s proposed guidelines, were the presence of fever on admission and during the C section [13]. Clark et al. suggested that cases with fever should be excluded in order to eliminate infectious causes of cardiovascular collapse seen in sepsis/systemic inflammatory response syndrome (SIRS) [13], but this patient was found to have negative blood cultures and no source of infection. We acknowledge Clark's much needed proposal of universal criteria for reporting research cases but see the limitations of these criteria in clinical practice and case presentations. The authors therefore would like to acknowledge this as an atypical presentation of AFE. This clinical diagnosis is supported by ruling out other likely diagnoses (see Table 5). The rapid return of functional status of pulmonary and cardiovascular systems are congruent with Leighton's findings in 2013 which similarly described the dramatic return of cardiovascular function with the use of A-OK in AFE [4, 14]. However, adding to the diagnostic uncertainty is the large dose of phenylephrine used in this case. Although not part of the AOK regime phenylephrine the dose used in this case may have contributed to resuscitation and hemodynamic stabilization rather than Atropine, Ondansetron, and Ketorolac.

**Figure 1:**
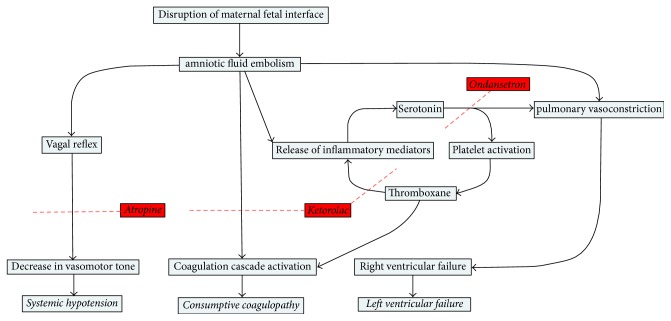
Proposed mechanism for Atropine, Ondansetron, and Ketorolac (A-OK) protocol.

**Figure 2:**
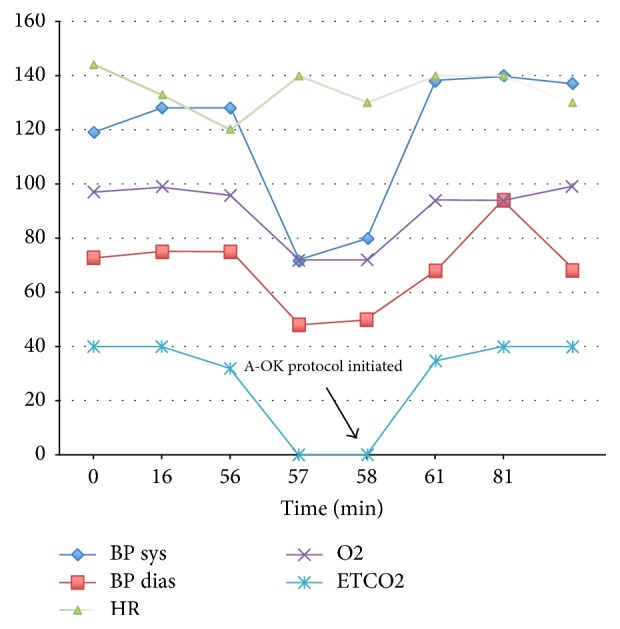
Graph of patient's vital signs prior and after initiation of A-OK therapy.

The current recommendations from the Society for Maternal Fetal Medicine suggest the use of Sildenafil, Dobutamine, Milrinone, inhaled nitrous oxide, Prostacyclin, and Norepinephrine when managing AFE [21]. Sympathomimetic medications help maintain blood pressure, but the mechanism of action of these agents does not address the potential underlying mechanisms of ventricular dysfunction. Historically, studies have suggested mechanical obstruction as the main mechanism for pulmonary hypertension [7]. However, more recent animal models have suggested serotonin and thromboxane act synergistically to cause platelet dysfunction, platelet degranulation, and pulmonary hypertension [14, 21, 22]. According to these models, pulmonary hypertension begins with serotonin stimulation of 5-HT receptors causing pulmonary vasoconstriction [21, 23]. Platelets are entrapped due to the pulmonary vasoconstriction and activated by thromboxane (TXA_2_) [14]. The thromboxane causes the recruitment and activation of additional platelets, compounding pulmonary hypertension with the release of more serotonin mediators causing a self-perpetuating cycle (Figure 1) [4, 14, 21]. Leañios et al. suggested these same mediators, while causing local vasoconstriction, cause systemic vagal stimulation leading to a fall in systemic vasomotor tone [21]. Finally, some animal models have suggested that consumptive coagulopathy occurs due to the activation of platelets, factor III, factor X, and amniotic fluid tissue factor leading to disseminated intravascular coagulation (DIC). However contradictory results have been reported [5, 7, 9].

Some authors have suggested the ventricular dysfunction is secondary to either pulmonary hypertension caused by serotonin and thromboxane or systemic hypotension caused by vagal stimulation [1, 11]. It has been proposed that Atropine and Ondansetron may act to block serotonin and vagal stimulation improving cardiovascular function rather than simply providing cardiovascular support [11]. Additionally, the A-OK regimen rather than replacing the consumed factors blocks the proposed cause of coagulopathy by inhibiting thromboxane with Ketorolac [1, 11] (see Table 6).

## 4. Summary

Traditionally the prognosis for AFE is poor, with a mortality rate ranging from 13 to 44% [1, 3]. Current recommended treatment for AFE includes pulmonary vasodilators, prostaglandins, sympathomimetics, and a host of other interventions [5, 10, 15, 21]. Along with these systemic mediators, large amounts of blood products such as FFP and PRBC are rapidly infused to combat DIC [1]. Other management methods such as bypass and exchange transfusion, by cardiovascular surgery, have also been reported in several cases [10, 16, 24–27].

The authors acknowledge that this case does not meet Clark's proposed criteria for AFE [12]. With AFE affecting many women across the globe and being a significant contributor to maternal mortality, efforts should be made to find effective treatments. We hope this case will prompt future investigation into novel treatments such as A-OK, which can be used in conjunction with traditional supportive measures.

## Figures and Tables

**Table 1:** 

Time/dose phenylephrine given
(1) 19:48: 200 mcg/ml
(2) 20:00: 200 mcg/ml
(3) 20:15: 400 mcg/ml
(4) 20:30: 400 mcg/ml
(5) 20:45: 400 mcg/ml

Total: 1800 mcg/ml

**Table 2:** Patient vital signs as demonstrated in Figure 2.

Event	Time	Minutes	EtCO_2_	RR	BP	Pulse	O_2_ saturation
Initial patient presentation to ED	11/29/16 16:59:00	0	NA	24	119/73	144	97% (room air)
	11/29/2016 19:15	16		21	128/50	133	99% (room air)

C-section procedure start	11/29/16 19:55:00	56	32	ETT	128/75	120	96% (on ETT)

Delivery of baby	11/29/16 19:56:00	57	0	ETT	72/48	140	72% (on ETT)

A-OK therapy initiated	11/29/16 19:57:00	58		ETT	80/50	130	94% (on ETT)

(Effect of) A-OK therapy	11/29/16 20:00:00	61	35	ETT	138/68	140	97% (on ETT)

Operating room timeout	11/29/16 21:20:00	81	37	21	140/94	140	94% (on ventilator)

**Table 3:** Risk factors and odd ratios for AFE. Abenhaim et al.

Risk factors	Odds ratio
Placenta previa	30.4
Preeclampsia	7.3
Cesarean section	5.7
Forceps delivery	4.3
Maternal age > 35 yrs	2.2
Vacuum delivery	1.9
All other methods of induction	1.5

**Table 4:** 

Proposed criteria for research reporting of amniotic fluid embolism [12]
(1) Sudden onset of cardiorespiratory arrest or both hypotension (systolic blood pressure < 90 mm Hg) and respiratory compromise (dyspnea, cyanosis, or peripheral capillary oxygen saturation [SpO2] < 90%)
(2) Documentation of overt DIC following appearance of these initial signs or symptoms, using scoring system of Scientific and Standardization Committee on DIC of the ISTH, modified for pregnancy Coagulopathy must be detected prior to loss of sufficient blood to itself account for dilutional or shock-related consumptive coagulopathy
(3) Clinical onset during labor or within 30 min of delivery of placenta
(4) No fever (38.0°C) during labor

**Table 5:** 

Differential diagnosis for AFE [5]
Pulmonary thromboembolism; more common later postpartum, chest CT was clear and lower limb Doppler was clear
Anesthetic complications; hypoxia was not associated with administration of any medication
Drug-induced allergic anaphylaxis; no rash or wheeze was observed
Myocardial infarction; no ECG changes and negative troponins
Cardiac arrhythmia; the intraoperative anesthesia record reports sinus tachycardia throughout monitoring
Aspiration of gastric contents; patient was had ETT tube inserted with cuff inflated preventing aspiration
Reaction to local anesthetic drugs; patients' condition deterioration does not correlate with any medications given
Sepsis: sepsis is ruled out since there was no source of infection, and patient had clear chest CT scan with SOB; there were no evidence of pneumonia, blood, and urine cultures which were negative

**Table 6:** Shamshirsaz and Clark in SOAP 2013 also describe this A-OK therapy with the addition of metoclopramide, which was not used in our patient [15]. The atropine is used to treat vagal overstimulation and improve vasomotor tone while Ondansetron blocks serotonin receptors inhibiting the release of further mediators [4, 16]. The Ketorolac blocks thromboxane production thereby preventing coagulopathy [4].

A-OK medication regimen [3]
Atropine 1 mg (vagolytic)
Ondansetron 8 mg (5-HT3 antagonist)
Ketorolac 30 mg (cyclooxygenase inhibitor)
